# Assessment of visual function under various lighting conditions in a cohort of active older drivers: dimensionality and principal metrics

**DOI:** 10.3389/fnins.2025.1511366

**Published:** 2025-04-15

**Authors:** Deyue Yu, Landon Perry, Thomas Kerwin, Jingzhen Yang, Zhong-Lin Lu

**Affiliations:** ^1^College of Optometry, The Ohio State University, Columbus, OH, United States; ^2^Driving Simulation Laboratory, The Ohio State University, Columbus, OH, United States; ^3^Center for Injury Research and Policy, Abigail Wexner Research Institute at Nationwide Children’s Hospital, Columbus, OH, United States; ^4^Division of Arts and Sciences, NYU Shanghai, Shanghai, China; ^5^Center for Neural Science and Department of Psychology, New York University, New York, NY, United States; ^6^NYU-ECNU Institute of Cognitive Neuroscience at NYU Shanghai, Shanghai, China

**Keywords:** visual function, mesopic vision, glare, aging, driving

## Abstract

**Purpose:**

While traditional driving ability evaluations typically assess visual acuity (VA) under photopic conditions, visual functions other than photopic VA also play a crucial role in driving. For older individuals, age-related vision change can impact driving abilities, particularly under mesopic lighting conditions with glare during nighttime driving. This study aims to investigate how visual functions vary across different lighting conditions, examine their correlations, and identify the principal visual function metrics that enable a more comprehensive assessment of active older drivers.

**Methods:**

Twenty active older drivers (aged 63 to 87 years; mean = 70 years) participated. All possessed valid driver’s licenses, drove at least once per week, and did not use any low vision aids for driving. Six participants had undergone cataract surgery. Participants completed a battery of visual tasks with their habitual correction for daily driving. VA, contrast sensitivity function (CSF) and visual field map (VFM) were measured under photopic and mesopic conditions using the qVA, qCSF and qVFM procedures. Additionally, VA and CSF were assessed in the presence of glare under mesopic condition. Correlations and principal component analysis (PCA) were conducted to identify principal visual function metrics.

**Results:**

VA and CSF exhibited variation across lighting conditions (*p*s < 0.005), with significant correlations observed between multiple pairs of visual functions. A trend of stronger correlations was found in participants who had undergone cataract surgery. PCA suggested that four metrics are necessary to explain most of the nonrandom variation in the data. Mesopic VA was the most informative measure, accounting for 47% of the total variance. Adding a measure of VFM increased the explained variance to 70%. To explain approximate 80% of the total variation, three measures were required, while four measures were needed to achieve 90%.

**Conclusion:**

Using a PCA-based selection approach, the minimal set of visual function metrics for evaluating visual function in active older drivers was identified. These findings provide valuable insights for establishing optimal clinical outcome measures for this population.

## Introduction

Drivers aged 65 or older have the highest rate of fatal nighttime crashes per mile driven among those older than 25 years (Cicchino and McCartt, 2014; Massie et al., 1995; Mortimer and Fell, 1989). Nighttime driving is inherently more demanding and hazardous compared to daytime driving because of reduced visibility caused by low light levels and glare (Wood, 2020). During nighttime driving, the visual environment is within the mesopic luminance range where the luminance levels are approximately 0.003–3 cd/m^2^. As detailed in a review by Wood (2020), visual function deteriorates under mesopic lighting conditions, which can be exacerbated by factors such as aging, visual impairment, and glare from road lighting and car headlights. Age-related declines in vision can significantly impact the driving abilities of older individuals, particularly under mesopic lighting conditions with glare during nighttime driving.

Conventional evaluations of vision for driving vary across countries and states, but typically focus on high contrast, photopic (daytime) visual acuity (VA) and sometimes include a basic photopic, peripheral visual field test. To qualify for driving, individuals need to have a visual acuity of 20/40 or better, with or without correction, and a horizontal visual field of 70° or more in each eye (The ECRI Institute, 2008). However, these evaluations may not fully capture a driver’s visual ability, as they do not assess important factors such as contrast sensitivity, low-light conditions, or glare. Research has shown that photopic VA alone is not a reliable predictor of driving ability for both nighttime (Gruber et al., 2013; Wood and Alfred, 2005) and daytime (Wood and Alfred, 2005) driving. This highlights the limitations of current driving-related vision evaluations and the need to incorporate additional vision tests to provide a more comprehensive assessment of active older driver’s visual function. The purpose of this study is to investigate how various visual functions vary across different lighting conditions, examine their correlations, and identify the principal visual function metrics that enable a more comprehensive assessment of active older drivers. This work represents a foundational step towards identifying appropriate vision tests and developing effective screening procedures for nighttime driving, especially for the elderly population. Achieving this ultimate objective will require extensive research into the driving abilities of older individuals and their practical fitness for safe nighttime driving.

To comprehensively characterize age-related vision changes in older drivers, it is essential to consider two additional metrics of functional vision: contrast sensitivity function (CSF) and visual field map (VFM). Contrast sensitivity (CS) is a critical aspect of vision that significantly influences overall visual performance. CS is often evaluated using a contrast sensitivity chart that measures CS at a particular target size or spatial frequency (Kiser et al., 2005; Pelli et al., 1988), though this may not fully capture variations in sensitivity across a range of spatial frequencies. In comparison, the CSF measures how sensitivity to contrast changes with different spatial frequencies (Hess and Howell, 1977; Jin et al., 2016; Jindra and Zemon, 1989; Pelli and Bex, 2013), and is considered a more accurate indicator of performance in everyday visual activities (Chung and Tjan, 2009; Owsley and Sloane, 1987; Stelmack and Massof, 2007), including driving (McGwin et al., 2000; Michael et al., 2009; Owsley et al., 2020; Puell et al., 2004; Rae et al., 2016; Swan et al., 2019). Another crucial measure that provides valuable information on functional vision is the VFM. Numerous studies have investigated the impact of VFMs on driving performance in older drivers. While the relationship between VFM and driving performance remains inconclusive on an individual level (Faraji et al., 2022), many studies demonstrate that visual field deficits can impair driving abilities even in mild to moderate stages (Huisingh et al., 2015; Wood et al., 2016). Specifically, the binocular visual field, which represents the overlap of visual fields from both eyes, is most relevant for driving as it contains crucial driving-related information primarily located within the central 30° of the visual field (Gruber et al., 2013). Driving performance can be significantly compromised when the binocular visual field is constricted to 40° or less (Wood and Troutbeck, 1992). At nighttime, the effective field of view is also constrained by the horizontal coverage (35° to 45°) of car headlights (Wilkinson and McGehee, 2019). Given these considerations, the present study focuses on examining the central 48° of the visual field.

Evidence suggests that mesopic vision deteriorates after the age of 40, particularly in the presence of glare (Gruber et al., 2013). Compared to young drivers, older drivers perform worse under low lighting conditions, primarily due to reductions in rod sensitivity, slower dark adaptation, reduced visual acuity, and increased sensitivity to glare (Andersen, 2012; Boot et al., 2013; Hertenstein et al., 2016; Jackson et al., 1999; Kaleem et al., 2012; Kimlin et al., 2017; Puell et al., 2004; Wood and Alfred, 2005). Additionally, many eye diseases that impair visual function are prevalent in the elderly population, such as cataracts and glaucoma, which can significantly affect nighttime driving ability (Janz et al., 2009; Owsley and McGwin, 1999). During nighttime driving, glare from bright artificial light sources such as oncoming headlights can cause discomfort and even temporary impairment on vision (Matesanz et al., 2024), significantly affecting a driver’s performance and safety (Kimlin et al., 2017). To date, few studies have examined the three metrics of functional vision simultaneously across multiple lighting conditions, including photopic, mesopic, and with the presence of glare, particularly in active older drivers. This gap in research underscores the need to explore how different lighting conditions impact visual functions in active older drivers and to determine the principal visual function metrics necessary for a comprehensive evaluation of visual function in this population.

In a recent preliminary study (Yang et al., 2024), we measured three basic visual functions (VA, CSF and VFM) and obtained various driving performance measures, including average speed, standard deviation of speed, standard deviation of lane position, and reaction time to visual stimuli, in active older drivers under three lighting conditions (photopic, mesopic, and mesopic with glare). The driving assessment was carried out using a high-fidelity driving simulator. Correlation analyses revealed distinct effects of VA, CSF and VFM on driving performance under different lighting conditions, indicating that visual functions had a greater impact on driving performance at night, particularly in the presence of glare. While these results do not suggest direct correlations between visual functions and real-world driving performance, they further underscored the need for and importance of comprehensive visual function assessments, especially under mesopic and glare conditions, to characterize age-related vision changes in older drivers. The present study focused on analyzing the extensive visual function data collected from the active older drivers under these three different lighting conditions (Yang et al., 2024). The goal was to explore the relationships among these visual function measurements and identify the minimal set of measures necessary for a thorough assessment of functional vision in active older drivers. Specifically, we used principal component analysis (PCA) as a selection tool to rank and select visual function metrics based on their contributions to the variation in visual function measures.

## Methods

### Participants

Study recruitment information was distributed through online volunteer directory/registry, social media postings, and flyers distributed at local community centers (Yang et al., 2024). Twenty active older drivers (9 females) aged 63 to 87 years (Mean ± SD: 70 ± 6 years) were recruited. All participants were English speakers, held a valid driver’s license, drove at least once per week, and did not use any low vision aids for driving. Based on self-report information, six of the participants (75 ± 7 years of age) had undergone cataract surgery. Among the remaining 14 participants (69 ± 4 years of age), one had been diagnosed with cataracts but had not undergone surgical intervention, one had dry eyes, and the rest had no history of eye disease. On average, the participants drove 5.2 days (SD = 1.6) per week, and 20.5 miles (SD = 11.3) and 43.75 min (SD = 22.9) per day on the day they drove. Each participant completed a battery of visual tasks with their habitual correction for daily driving. The research protocol was approved by the Nationwide Children’s Hospital Institutional Review Board (IRB), and all procedures complied with the Declaration of Helsinki. Informed consent was obtained from each participant prior to data collection.

### Apparatus

All tests were conducted using MATLAB (MathWorks Corp., Natick, MA, United States). The qVA test was displayed on a 24-inch Dell monitor (P2415Q) with a resolution of 3,840 × 2,160 pixels. For the qCSF test, a 46-inch NEC monitor (P463) with a resolution of 1,920 × 1,080 pixels was used. The qVFM test employed a Samsung 55-inch monitor (UN55FH6030) with a resolution of 1,920 × 1,080 pixels to display stimuli. Testing was performed in a room with no additional light, except for the testing screens and a glare source. The viewing distance was fixed at 4 meters for VA and CSF, and 30 cm for VFM, with participants using a chinrest to maintain consistent positioning. All tests were performed binocularly, consistent with real-life driving conditions where both eyes are typically used for visual tasks.

### Experimental design

VA, CSF and VFM, were assessed under both photopic and mesopic conditions in a dark room. Participants received a practice session before each test, conducted solely under the photopic condition. Prior to the beginning of both the photopic and the mesopic tests, participants underwent a minimum 5-min dark adaptation. In addition to standard mesopic testing, VA and CSF assessments were also conducted in the presence of glare. However, due to light reflection and spatial constraints between the participant and the testing screen, VFM measurements could not be obtained under the glare condition. For each lighting condition, high contrast VA was always measured first, followed by CSF. VFM evaluations were conducted last, under both photopic and mesopic conditions.

### Lighting conditions

The study involved three lighting conditions: photopic, mesopic, and mesopic with glare. Photopic vision relies on cones and occurs in well-lit conditions (luminance >3 cd/m^2^), such as daylight outdoors. Mesopic vision, occurring under low light conditions (0.003 cd/m^2^ < luminance <3 cd/m^2^), involves both rods and cones and is crucial for activities like nighttime driving. In this study, the background luminance of the test display was set to 9 cd/m^2^ or higher for the photopic condition. For the qVA, qCSF and qVFM tests, it was 84, 97 and 9 cd/m^2^, respectively. For the mesopic condition, the background luminance of test display was reduced either through a customized program or with neutral density filters. A luminance range of 0.1 and 1 cd/m^2^ has been shown to provide reliable and repeatable results for mesopic visual function measurements. Here, the luminance was reduced to 0.94, 1.00 and 0.47 cd/m^2^ for the qVA, qCSF and qVFM tests, respectively.

In the mesopic with glare condition (also referred to as glare condition), a Fiilex V70 lamp with a dome diffuser attached served as the glare source. The light was at eye level, facing the participants, and positioned 19 cm in front and 12 cm to the left of the midpoint between the two eyes to prevent obstruction of the testing screens. A color temperature of 3,000 K and the lowest intensity setting were used, providing a luminance of 5,983 cd/m^2^ and an illumination level of 305 lux at the midpoint between the two eyes. This illumination level is comparable to that of residential (30 to 300 lux) or office desk lighting (100 to 1,000 lux).

### Bayesian active learning

The qVA, qCSF and qVFM methods were used to measure VA, CSF and VFM, respectively. These methods, compared to conventional testing, assess the same aspects of visual function but with greater efficiency, precision and accuracy through advanced mathematical modeling. Specifically, the qVA, qCSF, and qVFM utilize the Bayesian active learning framework, as described by Lu and Dosher (2013), to efficiently assess visual function while maintaining high precision and accuracy in the measurements.

The methods integrate Bayesian inference with generative models of trial-by-trial responses to effectively capture patterns in visual function data. They also employ an information theoretic framework to select the most informative testing stimulus for each trial. With each trial, the estimation of the participant’s visual function is progressively refined based on the stimulus presented and the corresponding response provided.

In this study, each testing procedure was terminated after reaching a pre-determined, fixed number of trials. These efficient methods enabled us to collect multiple measurements of visual functions within a relatively short timeframe, which was essential for minimizing participant fatigue and ensuring sustained engagement throughout the study.

#### qVA

**Figure 1:**
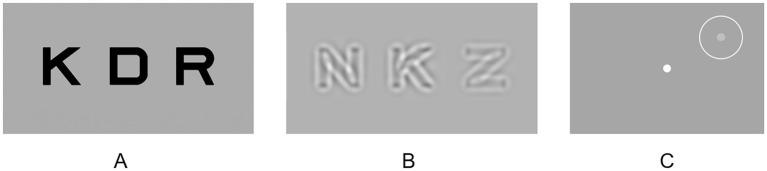
Examples of the testing stimuli in **(A)** qVA, **(B)** qCSF and **(C)** qVFM.

Each qVA test comprised 20 trials. In each trial, three high-contrast optotypes of the same size (Figure 1A) were randomly selected from the 10 Sloan letters (C, D, H, K, N, O, R, S, V, and Z). The size of the optotypes, determined by qVA (Lesmes and Dorr, 2019), varied from trial to trial. The stimuli remained on the screen until participant verbally identified all three letters.

#### qCSF

Each qCSF test comprised 30 trials. Within each trial, three equal-size bandpass-filtered optotypes were presented (Figure 1B). The stimulus size (center spatial frequencies) and contrasts for each trial were determined by the qCSF algorithm (Hou et al., 2015). Contrast varied among the three optotypes within each trial, with one of them near the estimated contrast threshold and two above it to ensure that they were not overly difficult for the participant to identify. The stimuli remained displayed on the screen until the participant verbally identified the exhibited letters. The area under log CSF (AULCSF) was used as summary metrics of CSF. The AULCSF was calculated by integrating the region beneath the log CSF curve (but >0) between the spatial frequencies of 1.5 to 18 cycles per degree.

#### qVFM

Each qVFM test contained 120 trials. Participants were instructed to maintain stable fixation at a fixation dot at the center of the display throughout the test. Each trial contained a beep sound, and a potential target (Figure 1C; a light disc with a diameter of 0.43° appeared at one of the 64 locations, evenly sampled across a visual field of 48° × 48° and cued with a circle, for 150 ms) (Xu et al., 2019). Participants pressed a key to report the presence or absence of the target using the qVFM algorithm. The visual field location and luminance of the target were adaptively adjusted from trial to trial. The volume under the surface of the VFM (VUSVFM), normalized to account for the variation of the background luminance, was used as a summary metric of VFM. The summary metric focused on relative visual field sensitivity rather than absolute values, allowing for a more equitable comparison of visual field performance across different background luminance levels.

### Data analysis

All visual function measurements, except for the two VUSVFM measures, followed normal distributions. A non-parametric method was used to evaluate the impact of lighting conditions on VUSVFM.

#### Correlations

The dimensionality of the visual function metrics was explored by assessing correlations among the eight visual function metrics measured across the three lighting conditions. Pearson correlations were reported here for all 28 pairs of outcomes to examine their linear relationships, which were evaluated with one-tailed, false discovery rate (FDR)-corrected *p*-values (Benjamini and Hochberg, 1995). This correlation analysis was also repeated for the subgroup of participants who had undergone cataract surgery.

#### Principal component analysis

Given the correlations among many of the visual function measures, principal component analysis (PCA) was employed to transform the eight visual function measures into principal components (non-correlated variables) to examine the dimensionality of the dataset and identify the most significant visual function metrics (Abdi and Williams, 2010; Bro and Smilde, 2014). Prior to performing PCA, the range of the visual function measures was standardized by transforming them to z-scores. The PCA was performed on a data matrix consisting of all 20 participants. The loadings of each visual function measure were examined for each principal component, along with the total variance explained by each component. Loading values, ranging from-1 to 1, reflect the contribution of each variable to the principal components. A loading value near-1 or 1 implies a strong influence of the variable on the principal component, whereas loadings near 0 suggest minimal influence.

#### Identifying the principal metrics of functional vision

To determine the minimal set of visual function metrics necessary for a comprehensive evaluation of visual function in older drivers, the visual function metrics were ranked based on their contributions to the explained variation in the data, using a PCA-based selection approach. This method involved five steps:

(1) A visual function measure of interest was manually selected as the first core metric. For instance, photopic VA could be chosen as the initial metric when evaluating an older driver’s functional vision. Subsequently, the contributions of other visual function measures were evaluated in addition to photopic VA.
(2) A linear regression was performed to establish the relationship between the selected initial metric (independent/explanatory variable) and each of the remaining visual function metrics (dependent variables). For instance, if photopic VA was chosen as the initial metric, linear regression was conducted to assess its relationship with each of the remaining seven visual function measures.
(3) The residuals of the regression model, representing the differences between the actual values and the values predicted by the model, along with the residual sum of squares (RSS), were calculated to examine the variation of each visual function measure that could not be explained by the initial metric. The total RSS is computed by summing RSS across visual function measures.
(4) PCA was performed on the residuals. By examining the coefficients/loadings, the residual visual function measures were ranked according to their contributions to the first principal component. The highest-ranked visual function metric, i.e., the metric with the highest loading, was then selected as the next core metric.
(5) Steps 2 to 4 were repeated on the residuals until all visual function metrics were selected.

This analysis provided insights into the optimal sequence of visual function measures to consider after the initial manually selected metric and how much variance each additional visual function measure explained. Each of the eight visual function metrics was evaluated as the initial core metric in this manner.

## Results

### Visual function measures across the three lighting conditions

**Figure 2:**
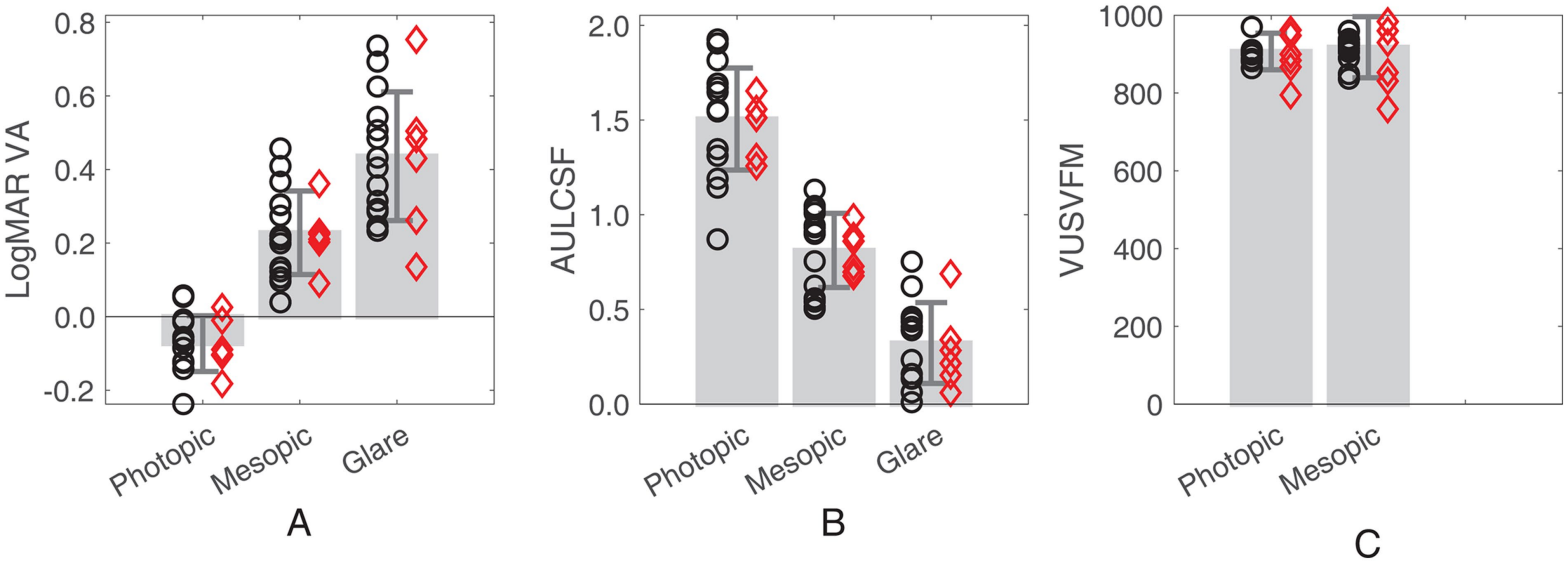
Bar plots showing **(A)** LogMAR VA, **(B)** AULCSF and **(C)** VUSVFM measured under the three lighting conditions (photopic, mesopic, and mesopic with glare). Error bars represent ± standard deviation. Red diamonds represent the individuals who had cataract surgery. Black circles represent the individuals who did not have cataract surgery.

As shown in Figure 2 and Table 1, both VA (*F* (2,38) = 144, *p* < 0.005) and AULCSF (*F* (2,38) = 362, *p* < 0.005) exhibit variations across lighting conditions. Both measures decrease with a shift from photopic to mesopic conditions and deteriorate further in the presence of glare under the mesopic condition. Conversely, for VUSVFM, comparable performance is attained between the photopic and mesopic conditions (*p* = 0.33; Wilcoxon signed rank test). Although better visual field sensitivity is generally expected with the reduction of background luminance from photopic to mesopic levels, the absence of a significant difference between these two conditions is not surprising. This is because the VUSVFM measure is a normalized value, adjusted for variations in background luminance.

**Table 1:** Visual function measures (LogMAR VA, AULCSF and VUSVFM) under the three lighting conditions (mean ± standard deviation).

	Photopic	Mesopic	Mesopic with glare
LogMAR VA	−0.07 ± 0.08	0.23 ± 0.11	0.44 ± 0.17
AULCSF	1.51 ± 0.27	0.81 ± 0.20	0.32 ± 0.21
VUSVFM	907 ± 47	917 ± 79	/

### Correlations

Table 2 illustrates significant correlations among multiple pairs of visual function outcomes. Firstly, excluding VA between the photopic and glare conditions, all three visual functions exhibited significant correlations across different lighting conditions (see the light gray cells; *r* = 0.47 to 0.92; *p*s ≤ 0.04). Secondly, VA and AULCSF were consistently correlated across all three lighting conditions (see the dark gray cells; *r* = −0.90 to −0.50; *p*s ≤ 0.03). Additionally, VAs measured in the mesopic condition, with and without glare, correlated significantly with AULCSF measured under any of the three lighting conditions (*r* = −0.90 to −0.52; *p*s ≤ 0.02; Table 2). Lastly, no correlation was found between VUSVFM and VA or between VUSVFM and AULCSF. Upon re-examination of the correlations within the subgroup of participants who had undergone cataract surgery, a trend of stronger relationships was observed. Although in many cases, the *p*-values from the cataract surgery subgroup did not reach the critical value for statistical significance, possibly because of the smaller sample size, the correlation coefficients were almost consistently higher for the subgroup compared to the entire cohort (Table 2).

**Table 2:** Correlation coefficients for all 28 pairs of outcomes across different conditions and tests for both the whole group (black texts) and the subgroup with cataract surgery (red texts).

		LogMAR VA		AULCSF			VUSVFM	
		Mesopic	Glare	Photopic	Mesopic	Glare	Photopic	Mesopic
LogMAR VA	Photopic	0.61*	0.27	−0.50*	−0.39	−0.26	−0.29	−0.22
		0.86	0.84	−0.64	−0.59	−0.92*	−0.70	−0.80
	Mesopic	/	0.66*	−0.86*	−0.90*	−0.58*	−0.19	−0.22
		/	0.95*	−0.77	−0.77	−0.89	−0.58	−0.65
	Glare	/	/	−0.52*	−0.63*	−0.86*	−0.05	−0.01
		/	/	−0.53	−0.65	−0.88	−0.62	−0.69
AULCSF	Photopic	/	/	/	0.90*	0.47*	0.29	0.31
		/	/	/	0.83	0.62	0.38	0.42
	Mesopic	/	/	/	/	0.56*	0.16	0.20
		/	/	/	/	0.72	0.39	0.50
	Glare	/	/	/	/	/	0.01	−0.03
		/	/	/	/	/	0.48	0.63
VUSVFM	Photopic	/	/	/	/	/	/	0.92*
		/	/	/	/	/	/	0.97*

^*^One-tailed, FDR-corrected *p*-value < 0.05. Light gray cells contain the correlations among different lighting conditions for each visual function. Dark gray cells contain the correlation between VA and AULCSF for each lighting condition.

### Principal component analysis

Table 3 presents the loadings of all eight visual function measures for each of the principal components. A criterion loading value (0.35) was calculated, representing the loading value when all variables contribute equally to the principal component. A variable with a loading greater than the criterion value is deemed as an important contributor to the principal component, as it contributes more than one variable’s worth of information. As shown in Table 3, using PC1, PC2, PC3 and PC4 collectively accounted for 95% of the total variation in the data. Bartlett’s test confirmed that four dimensions were necessary to explain the nonrandom variation in the data. In essence, an accurate representation of the data could be constructed using the first four PCs.

**Table 3:** Loadings of all eight visual function measures for each of the principal components, and percentage of the total variance explained by each principal component.

		PC1	PC2	PC3	PC4	PC5	PC6	PC7	PC8
LogMAR VA	Photopic	0.30	−0.13	**0.58**	**0.70**	−0.03	−0.05	−0.05	−0.25
	Mesopic	**0.46**	0.06	0.22	−0.09	0.31	0.48	0.17	0.61
	Glare	**0.37**	0.28	**−0.44**	0.21	0.61	−0.37	0.13	−0.16
AULCSF	Photopic	**−0.44**	0.04	−0.22	**0.38**	0.37	0.59	−0.36	−0.09
	Mesopic	**−0.44**	−0.09	−0.11	**0.45**	−0.04	−0.20	0.57	0.47
	Glare	−0.35	−0.30	**0.48**	−0.31	0.63	−0.25	0.02	−0.05
VUSVFM	Photopic	−0.17	**0.63**	0.27	−0.11	0.00	0.26	0.51	−0.39
	Mesopic	−0.17	**0.64**	0.24	0.07	−0.02	−0.33	−0.48	0.39
% of the total variance		53%	24%	11%	8%	2%	1%	1%	1%

The columns are arranged in descending order according to the component variance. Only the first four principal components are significant. The most important loadings (loading value >0.35) in the first four principal components are highlighted in boldface.

### Identifying the principal metrics of functional vision

**Figure 3:**
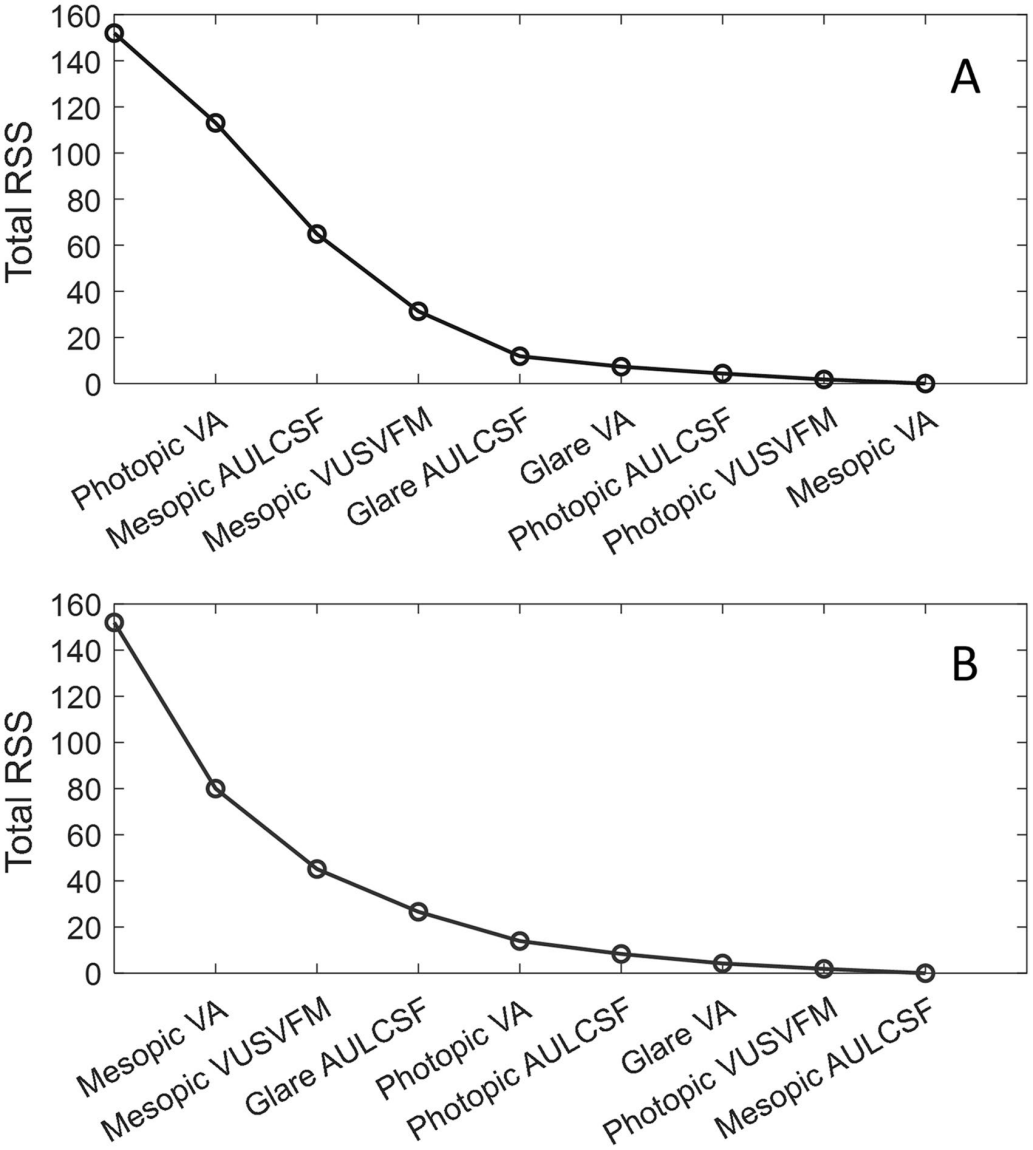
Examples of reduction of the total RSS with each additional core metric selection. **(A)** Photopic VA was manually selected as the first core metric. **(B)** Mesopic VA was manually selected as the first core metric. The first data point on the left corresponds to the initial total RSS when none of the visual function metrics were considered.

Using the PCA-based selection method, we evaluated and ranked the eight visual function measures according to their contributions to explain the variance in visual function measures Figure 3 shows two examples on how the total RSS reduced from 152 to 0 with each additional core metric selected.

To determine the principal visual function metrics necessary for a comprehensive evaluation of visual function in older drivers, the goodness-of-fit is evaluated by the the coefficient of determination (*r*^2^; see Equation 1) that measures the percent variance accounted for by the model consisting of the selected metrics (components). A higher *r*^2^ value represents smaller differences between the observed ( $y_{\mathrm{observed}}$ ) and the predicted ( $y_{\mathrm{predicted}}$ ) values.


(1) $$r^{2}=1-\frac{\sum_{m=1}^{8}\left(\sum_{n=1}^{20}{\left(y_{\mathrm{observed}}-y_{\mathrm{predicted}}\right)}^{2}\right)}{\sum_{m=1}^{8}\left(\sum_{n=1}^{20}{\left(y_{\mathrm{observed}}-y_{\mathrm{mean}}\right)}^{2}\right)}$$

where $y_{\mathrm{mean}}$ is the group mean of a visual function measure.

Table 4 shows the *r*^2^ values associated with each addition of a core metric. As shown in Table 4, three visual function metrics were required to attain an approximate 80% coefficient of determination, and four metrics were needed to achieve 90%. If only one measure of visual function can be obtained, the best metric to use would be mesopic VA. This metric alone explained 47% of the overall variance. If two measures of visual function can be collected, the most informative pairs of metrics (together explaining 70% of the total variation) would always contain mesopic VA and a measure of VFM (photopic or mesopic). When there are three visual function measures, the best metric combinations would consistently include one measure of VFM and at least one measure of CSF. In most cases, the first three metrics included one measurement for each of the three visual functions and each of the three lighting conditions, which collectively accounted for around 80% of the overall variation in visual function scores.

**Table 4:** Lists of visual function metrics ranked with respect to their contributions to the coefficient of determination for each pre-selected, first core metric (Metric 1).

	Metric 1	Metric 2	Metric 3	Metric 4	Metric 5	Metric 6	Metric 7	Metric 8
	Photopic VA	Mesopic AULCSF	Mesopic VUSVFM	Glare AULCSF	Glare VA	Photopic AULCSF	Photopic VUSVFM	Mesopic VA
*r* ^2^	26%	57%	79%	92%	95%	97%	99%	100%
	Mesopic VA	Mesopic VUSVFM	Glare AULCSF	Photopic VA	Photopic AULCSF	Glare VA	Photopic VUSVFM	Mesopic AULCSF
*r* ^2^	47%	70%	82%	91%	95%	97%	99%	100%
	Glare VA	Mesopic VUSVFM	Photopic AULCSF	Photopic VA	Mesopic VA	Glare AULCSF	Photopic VUSVFM	Mesopic AULCSF
*r* ^2^	37%	63%	81%	91%	94%	97%	99%	100%
	Photopic AULCSF	Mesopic VUSVFM	Glare AULCSF	Photopic VA	Glare VA	Mesopic VA	Photopic VUSVFM	Mesopic AULCSF
*r* ^2^	43%	65%	81%	91%	95%	97%	99%	100%
	Mesopic AULCSF	Photopic VUSVFM	Glare AULCSF	Photopic VA	Glare VA	Photopic AULCSF	Mesopic VUSVFM	Mesopic VA
*r* ^2^	44%	68%	81%	92%	95%	97%	99%	100%
	Glare AULCSF	Mesopic VUSVFM	Photopic AULCSF	Photopic VA	Glare VA	Mesopic VA	Photopic VUSVFM	Mesopic AULCSF
*r* ^2^	34%	60%	81%	91%	95%	97%	99%	100%
	Photopic VUSVFM	Mesopic VA	Glare AULCSF	Photopic VA	Photopic AULCSF	Glare VA	Mesopic VUSVFM	Mesopic AULCSF
*r* ^2^	26%	70%	83%	91%	95%	97%	99%	100%
	Mesopic VUSVFM	Mesopic VA	Glare AULCSF	Photopic VA	Photopic AULCSF	Glare VA	Photopic VUSVFM	Mesopic AULCSF
*r* ^2^	26%	70%	82%	91%	95%	97%	99%	100%

The coefficient of determination (*r*^2^) measures percent variance accounted for by the model consisting of the selected metrics.

## Discussions and conclusions

Aging, even in the absence of ocular disease, is associated with reduced mesopic vision and increased glare sensitivity (Andersen, 2012; Hertenstein et al., 2016; Kaleem et al., 2012; Kimlin et al., 2017; Puell et al., 2004; Wood and Alfred, 2005). With the growing number of older drivers, the likelihood of nighttime driving crashes is anticipated to rise, posing significant challenges to road safety (National Highway Traffic Safety Administration, 2018; Allen et al., 2019; Cicchino and McCartt, 2014). Understanding the specific vision factors contributing to nighttime driving difficulties in older drivers is crucial for developing effective vision screening procedures and providing appropriate support to mitigate related issues. While this study did not assess driving performance, it represents an initial step towards this important goal. By examining visual function data collected using efficient testing methods under various lighting conditions, this study explored the dimensionality of the metrics, and identified the principal metrics that were crucial for comprehensive assessments of visual function in active older drivers. Our findings suggest that, depending on the specific needs, criteria for variance interpretation, and limitations on testing time and resources, different subsets of visual function metrics may be more suitable for practical use.

While VA, CSF and VFM obtained in the different lighting conditions all provide valuable information about visual function in active older drivers, we employed principal component analysis to assess the contributions of various metrics and identify the most informative ones. Conventional PCA indicated that four principal components could capture most of the variance in the data. However, we aimed not only to reduce the dimensionality of the data but also reduce the number of metrics needed for practical application. Using the PCA-based selection method, we ranked the eight visual function measures, providing insights into which metrics should be prioritized in sequence for the most informative evaluation.

Although photopic VA is commonly used in conventional vision evaluation for driving, it has limited sensitivity in predicting nighttime (Gruber et al., 2013) and daytime (Wood and Alfred, 2005) driving ability, and does not fully capture the complexity of functional vision under different lighting conditions. In fact, among the eight visual function measurements obtained in this study, photopic VA was one of the least significant predictors in predicting functional vision in older drivers. For instance, photopic VA did not even exhibit a significant correlation with VA in the mesopic condition with glare. If we want to obtain a visual function metric in addition to photopic VA to provide more information about the visual function of active older drivers, according to our findings, the best option would be mesopic CSF. Together, the two measurements accounted for 57% of the total variance. If we are limited to obtaining only one measure of visual function, the optimal metric to employ would be mesopic VA which by itself accounted for 47% of the total variance. This is also confirmed by the principal component analysis which showed that mesopic VA has the highest loading (contribution) to the first principal component. Previous research has also demonstrated that mesopic VA is more pertinent to older people’s night driving abilities than photopic VA (Gruber et al., 2013).

Compared to conventional methods, the qVA, qCSF and qVFM procedures are much more efficient while offering high accuracy and precision (Hou et al., 2015; Lesmes and Dorr, 2019; Xu et al., 2019). Using these active learning procedures, it typically takes normally sighted young adults about 2, 3 and 4 min to complete one measure of VA (20 trials), CSF (30 trials) and VFM (120 trials), respectively. Depending on individual differences, some of the older participants took a similar amount of time to complete these measurements while others spent a little longer to provide their responses to stimuli close to their thresholds. Although it takes much less time to measure CSF and VFM with the active learning procedures compared to the conventional methods, VA remains to be the easiest and quickest assessment among the three because it requires the fewest trials and has no requirement on maintaining stable fixation.

Given the time and effort considerations, the most efficient metric or metric combination for evaluating the visual function of older drivers should be the one that contains only VA tests or the most VA tests. For instance, if three measurements can be acquired, to maximize the coefficient of determination, the recommendation for measurement would always include one measure of VFM and one or two measures of CSF. Since VA takes much less time to measure, it may be best to select one VA and one CSF measurement instead of two CSF measurements. If using *r*^2^ = 90% as a criterion, four visual function measurements are required. It may be more time efficient to adopt a set with two VA measurements. When the available evaluation time is unclear, it may be the best practice to start with mesopic VA, the most informative and fastest measure. In instances where glare testing is not feasible, whether due to setup constrains or participant discomfort, sequence of photopic VA, mesopic CSF, and mesopic VFM (see Table 4) may be the optimal choice, capturing nearly 80% of the overall variation in visual function data. Our ranking results can be used to guide evaluator to efficiently assess visual function in older drivers. By prioritizing the most informative visual function measures early on, evaluators can gather valuable data while minimizing participant fatigue and maximizing evaluation accuracy. Our findings provide insights into how to optimize and streamline the evaluation process and ensure that older drivers receive comprehensive and targeted assessments of their visual function. Importantly, our study did not assess real-world driving performance, and further research is necessary to determine how these visual function metrics impact individual driving abilities.

Notably, when the starting metric was a CSF measure, VA did not possess sufficient new information to be ranked among the top three metrics (Table 4). This may be because a full CSF contains a measure of VA (the high-frequency cutoff of CSF). In qCSF, the high-frequency cutoff corresponds to the spatial frequency at which contrast sensitivity is 2.0 (i.e., contrast threshold = 0.5) (Hou et al., 2010). Indeed, there was a strong correlation between the high-frequency cutoff of CSF and the VA obtained from the qVA test (*r* = −0.96, *p* < 0.005). When calculating AULCSF, the area under the log CSF curve was integrated within the spatial frequency range of 1.5 to 18 cycles per degree. The high-frequency cutoffs in most conditions and participants fell within this range. In other words, AULCSF, most of the times, contains VA information and beyond, which explains the association between AULCSF and VA and why a VA measure following an AULCSF measure did not make a substantial contribution compared to non-VA measurements. This shows that if we perform a CSF test first, we may not need to measure VA unless more than 80% coefficient of determination is required.

Cataracts are a leading cause of visual impairment in adults over the age of 60 (Klein et al., 1992), resulting in reduced vision and increased glare sensitivity. A previous study revealed that drivers with cataracts had a markedly higher crash rate compared to those without, and that this rate could be reduced by half after cataract surgery (Owsley et al., 2002). Here we explored how cataract surgery might influence the relationships among visual function measures. We hypothesized that intraocular lenses outperform natural aging lenses (which may or may not have cataract) by providing more uniform light transmittance across the visual field. This improvement in light transmission could reduce task-dependent noise and strengthen the correlations between visual function measurements. As expected, we found a trend toward stronger correlations between visual function outcomes in the subgroup of participants who had undergone cataract surgery. Out of the six participants in the subgroup, five underwent bilateral cataract removal surgery, and the other participant received cataract surgery, however, the specific eyes involved were unknown. No other detailed information was collected about their surgeries and interocular lenses (e.g., tinted vs. non-tinted; monofocal vs. multifocal and other types). The testing stimuli in the qVA, qCSF, and qVFM tests differ by size and retinal location. While it is unclear whether the participants in the non-surgery group exhibited any degree of cataract, here is one potential explanation. Comparing to the intraocular lenses in the participants who had cataract surgery, the lenses in the participants who did not undergo cataract surgery may have less uniform transmittance. A possible consequence is that the varying transmittances at different parts of the lenses have different impacts on various visual function measures in the non-surgery group, resulting in overall lower correlations when evaluating the entire group. With stronger correlations in the surgery group, fewer visual function metrics may be required to explain the majority of the variance in the data. Additional research with a larger sample size and more detailed information on the cataract surgeries, interocular lenses and cataract status in the non-surgery group could help confirm and better understand the impact of cataract surgery on visual function relationships.

While the findings of this study provide valuable insights, it has several limitations: (1) The study analyzed the data from 20 active older drivers. The sample-to-variable ratio is low with eight visual function measures, potentially lack sufficient statistical power to yield stable, generalizable PCA results (Osborne and Costello, 2004). Additionally, the subject recruitment source is limited. Further studies with a larger, more diverse, and representative sample are needed to confirm these findings and ensure generalizability of the results. (2) Although motion perception or sensitivity to motion may influence driving performance (Henderson and Donderi, 2005; Lacherez et al., 2014), this study focuses solely on basic visual function metrics using static stimuli. (3) The Useful Field of View (UFOV), the area from which an individual can extract visual information in a single fixation (Ball and Owsley, 1993), decreases with age and strongly correlates with on-road driving performance in older drivers (Willstrand et al., 2017). Including UFOV measurements could have altered the PCA structure and affected the identified principal visual function metrics.

The present study explored the relationships among visual function measurements and, using a PCA-based selection approach, identified the principal visual function metrics essential for a comprehensive evaluation of visual function in older drivers. In practice, it may be beneficial to prioritize VA measurements, given that among the eight visual function metrics, only photopic VA and, in some cases, a basic photopic peripheral visual field test are currently used by the Department of Motor Vehicles to assess driver’s license eligibility. The visual function measures employed in this study are straightforward to implement and could enhance the accuracy of driver’s license eligibility assessments, especially when there is a demonstrated need and when the benefits of implementing additional assessments outweigh the associated costs and burdens. These findings lay the groundwork for future studies, providing valuable insights for establishing optimal clinical outcome measures for active older drivers. However, future studies are needed to assess how these visual function metrics influence real-world driving behaviors and to determine their validity and reliability in predicting individual driving safety and performance. Additionally, another potential approach to translate these findings into real-world solutions is through the improvement of headlights, street lighting, road signs and markings, vehicle technologies, and other environmental factors to better accommodate the visual needs of older drivers.

## Data Availability

The data supporting the conclusions of this article are available by request from the corresponding author.
